# Supplementary material for: Biocontainment strategies for in vivo applications of Saccharomyces boulardii
Source: Front Bioeng Biotechnol. 2023 Feb 20;11:1136095. doi: 10.3389/fbioe.2023.1136095 (PMC9986445; doi:10.3389/fbioe.2023.1136095)
Supplement: Supplementary file 1 [file DataSheet1.pdf]

Supplementary Material

Biocontainment strategies for *in vivo* applications of *Saccharomyces boulardii*

Karl Alex Hedin, Vibeke Kruse, Ruben Vazquez-Uribe*, Morten Otto Alexander Sommer*

*Corresponding authors
Morten Otto Alexander Sommer (msom@bio.dtu.dk)
Ruben Vazquez-Uribe (ruvas@biosustain.dtu.dk)

Supplementary Figures and Tables

Supplementary Figures

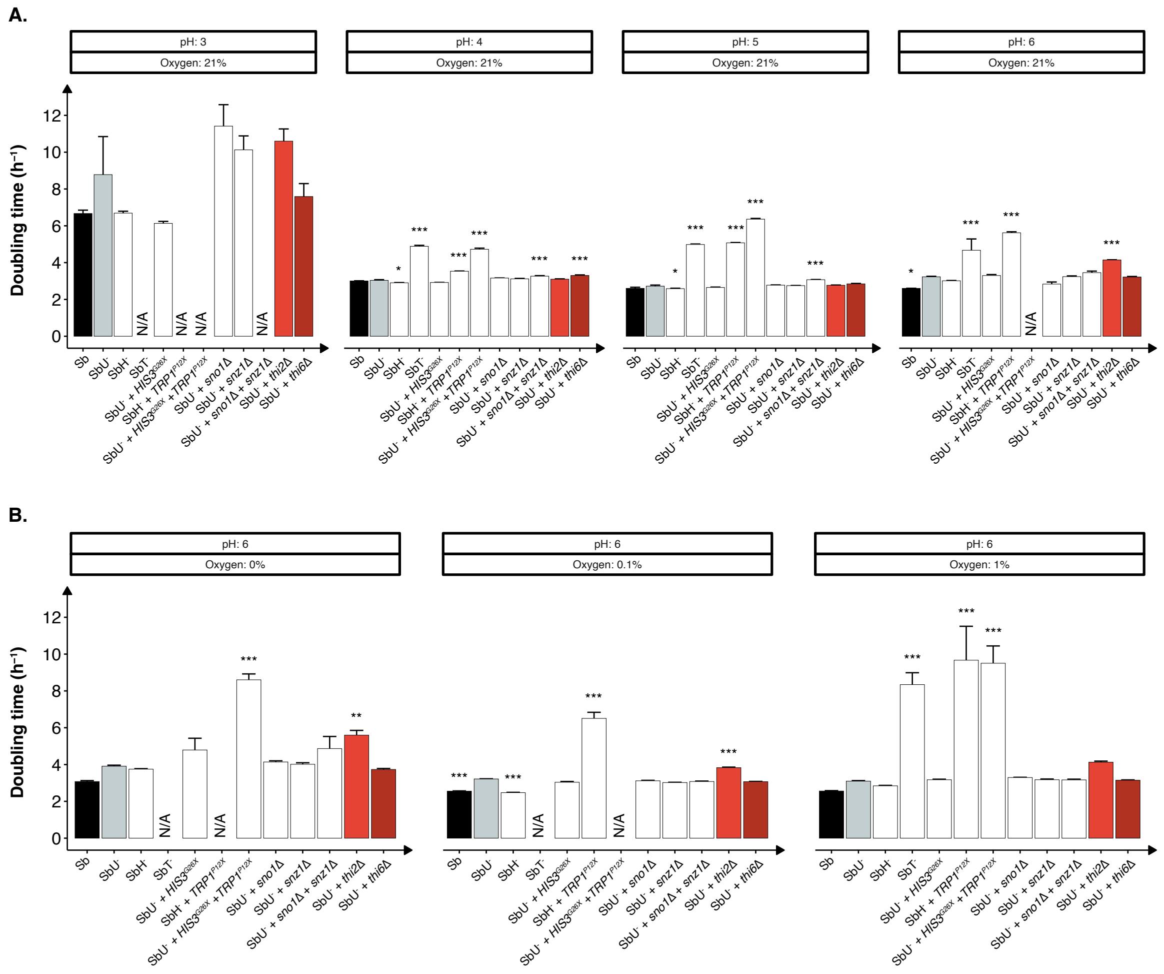

Supplementary Figure S1. Growth characterisation of different auxotrophic strains of *S. boulardii*. (A) Bar plot of the mean doubling time (h^{-1}) in pH 3, 4, 5 and 6 at aerobic cultivation. **(B)** Bar plot of the mean doubling time (h^{-1}) under anaerobic (0 %) and microaerobic (0.1 % and 1 %) at pH 6. Data presented as mean + SEM (n = 3). One-way ANOVA, Dunnett's post hoc test with Sb *URA3*^{S81X} as reference.

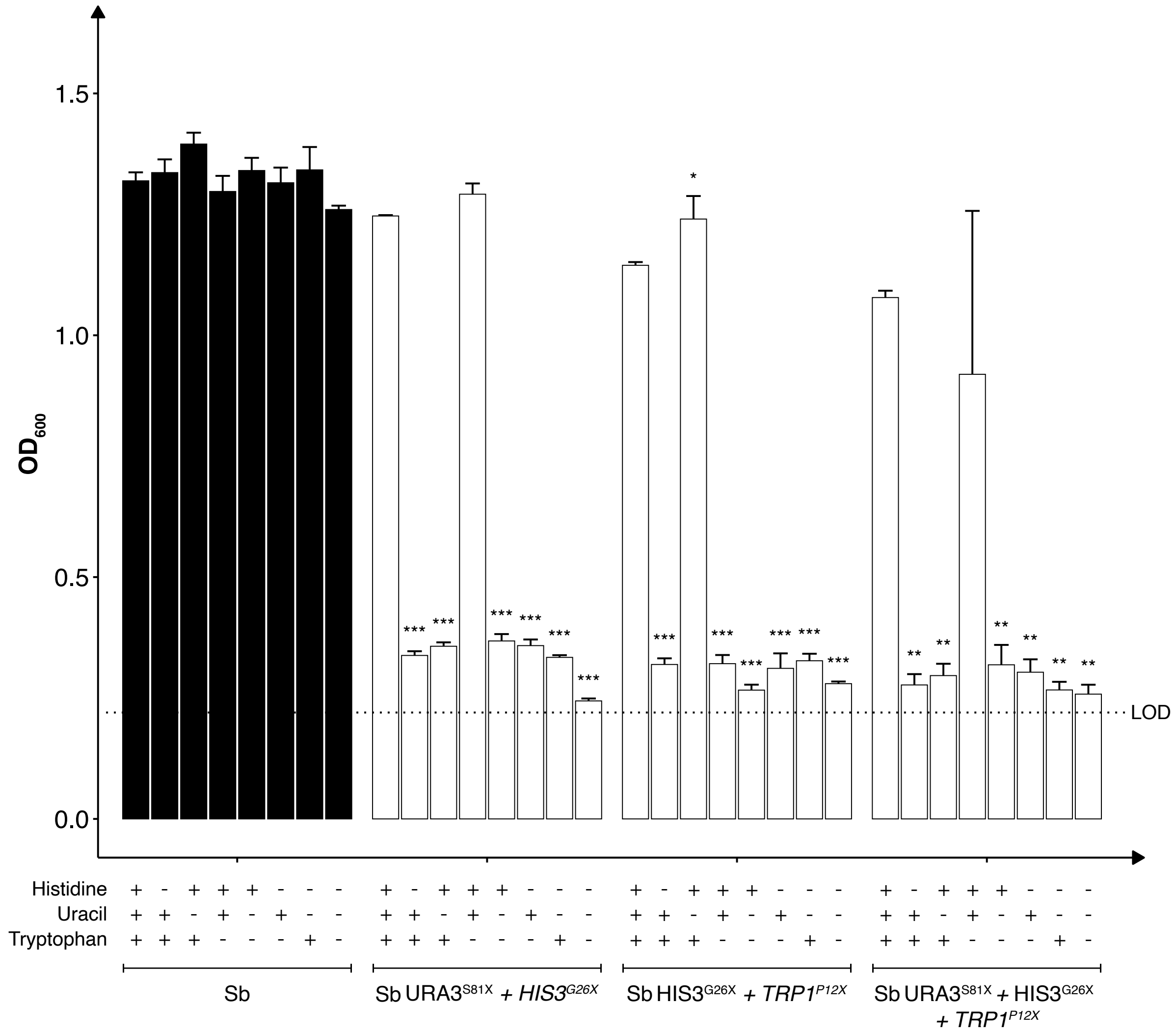

Figure S2. Growth characterisation of double and triple gene disruptions *S. boulardii*. Bar plot of mean OD_{600} after 120 hours with and without the required nutrition supplemented. Limit of detection (LOD). Data presented as mean + SEM (n = 3). * $p < 0.05$, ** $p < 0.01$ and *** $p < 0.001$. One-way ANOVA, Dunnett's post hoc test for each strain with fully supplemented media as reference.

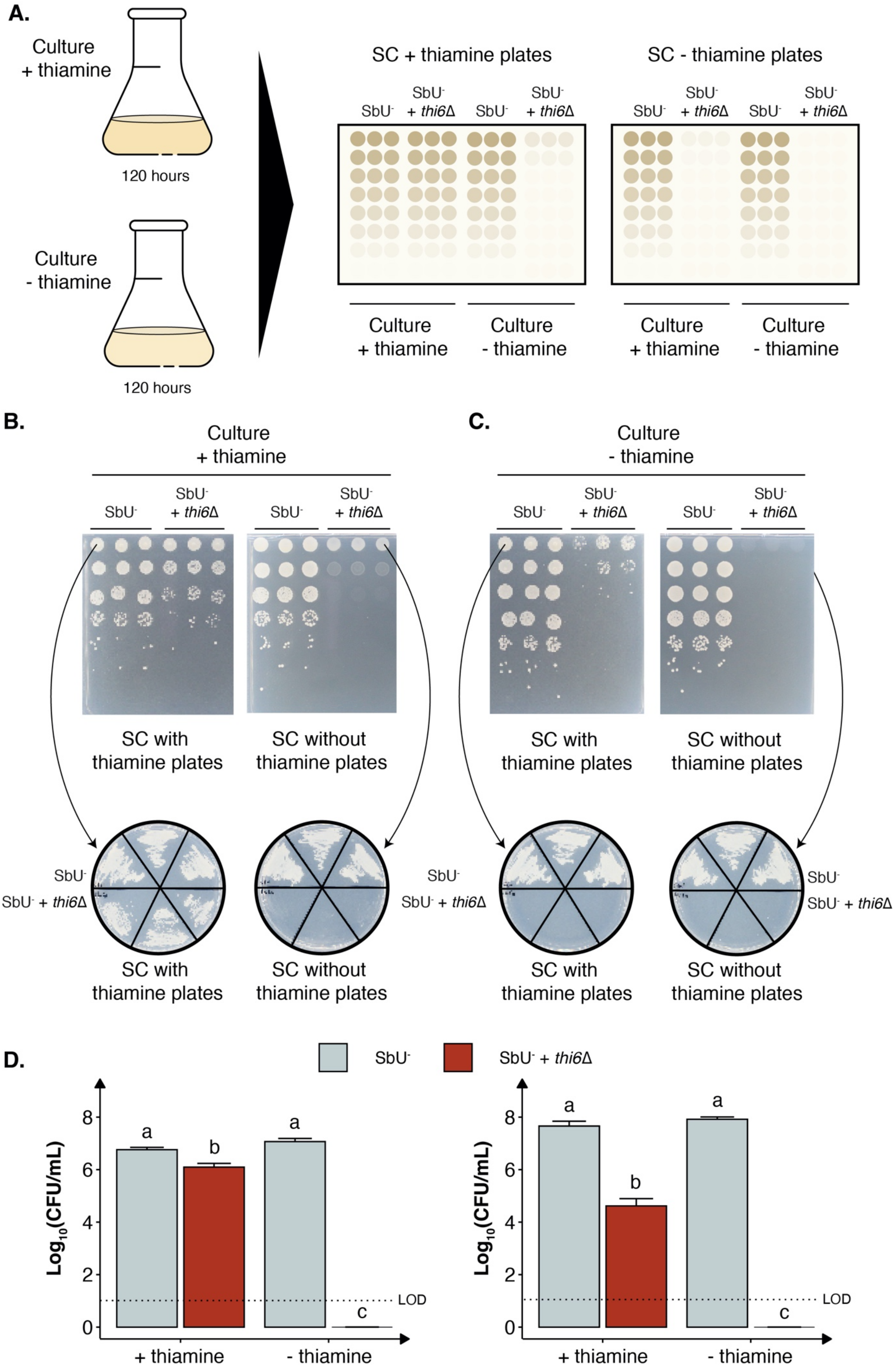
A.
Culture
+ thiamine
120 hours
Culture
- thiamine
120 hours
SC + thiamine plates
SC - thiamine plates
SbU$^-$
SbU$^-$ + *thi6Δ*
Culture
+ thiamine
Culture
- thiamine
B.
Culture
+ thiamine
C.
Culture
- thiamine
SC with
thiamine plates
SC without
thiamine plates
SbU$^-$
SbU$^-$ + *thi6Δ*
D.
SbU$^-$
SbU$^-$ + *thi6Δ*
Log_{10}(CFU/mL)
0
2
4
6
8
a
b
c
LOD
+ thiamine
- thiamine

Supplementary Figure S3. Escape rate assay of the *thi6*Δ *S. boulardii* strain. (A) Graphical illustration of the experimental design. The *S. boulardii* strains were cultivated for 120 hours in SC with (400 µg/mL) and without thiamine at 37°C. The cultures we spun down and resuspended in 500 µL and spotted on SC plates with and without thiamine. All plates were incubated for 72 hours at 37°C. **(B)** The serial dilution and re-streak of SbU^- and SbU^- + *thi6*Δ cultivated in SC with 400 µg/mL thiamine. **(C)** The serial dilution and re-streak of SbU^- and SbU^- + *thi6*Δ cultivated in SC without thiamine. Grown biomass from the undiluted samples were spread out on fresh selection plates to verify potential escapers. **(D)** The log_{10} CFU/mL from the serial dilution spotting. Data presented as mean + SEM ($n = 3$). Two-way ANOVA, Tukey post hoc test. The different letters (a, b, and c) above the bars indicate statistically different groups (significance level at $p < 0.05$)

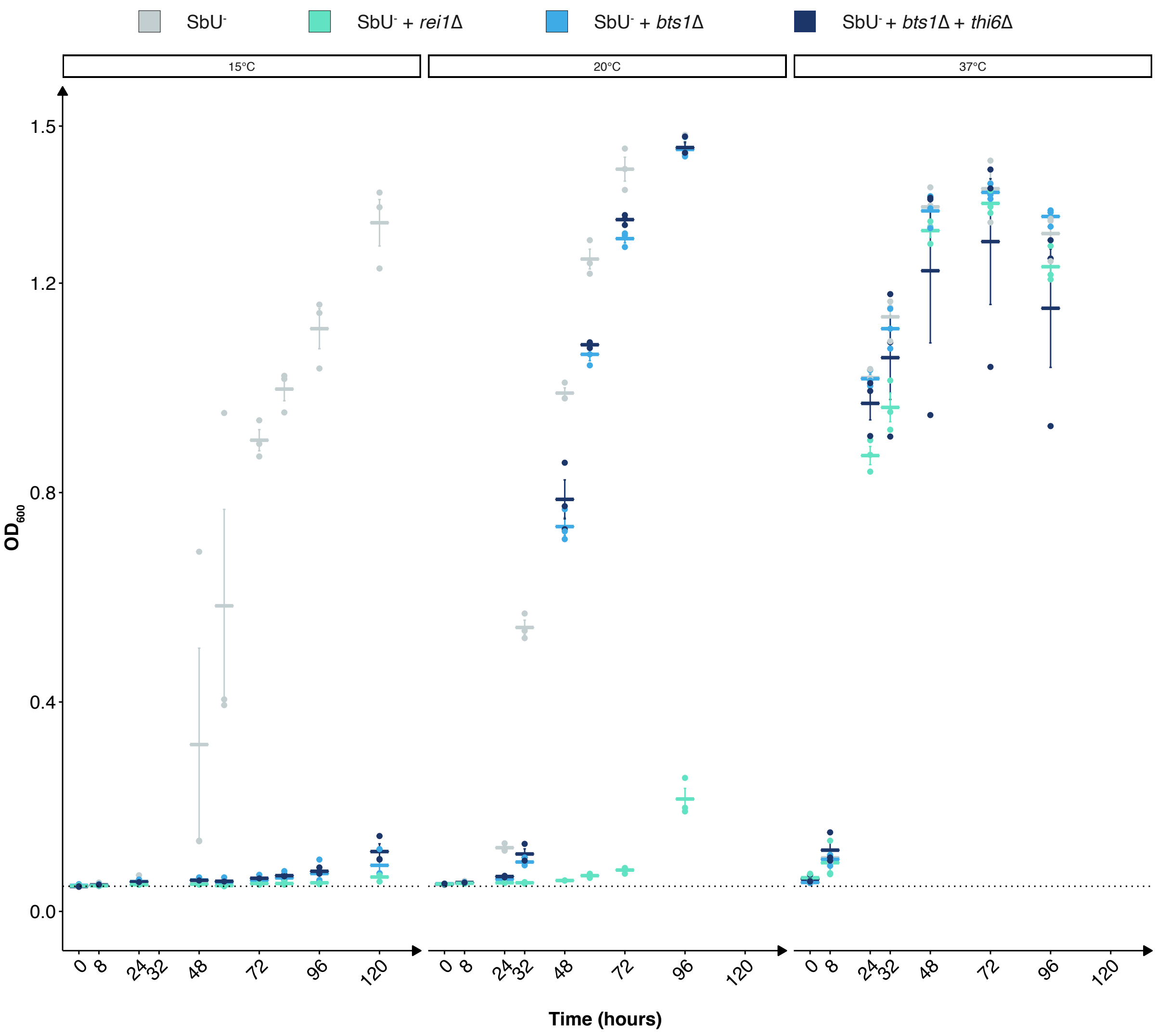

Supplementary Figure S4. Growth performance of the cold-sensitive strains at different temperature. The mean OD_{600} over time at different temperature (15 °C, 20 °C and 37 °C). Data presented as mean ± SEM (n =3). Each point represents a biological replicate.

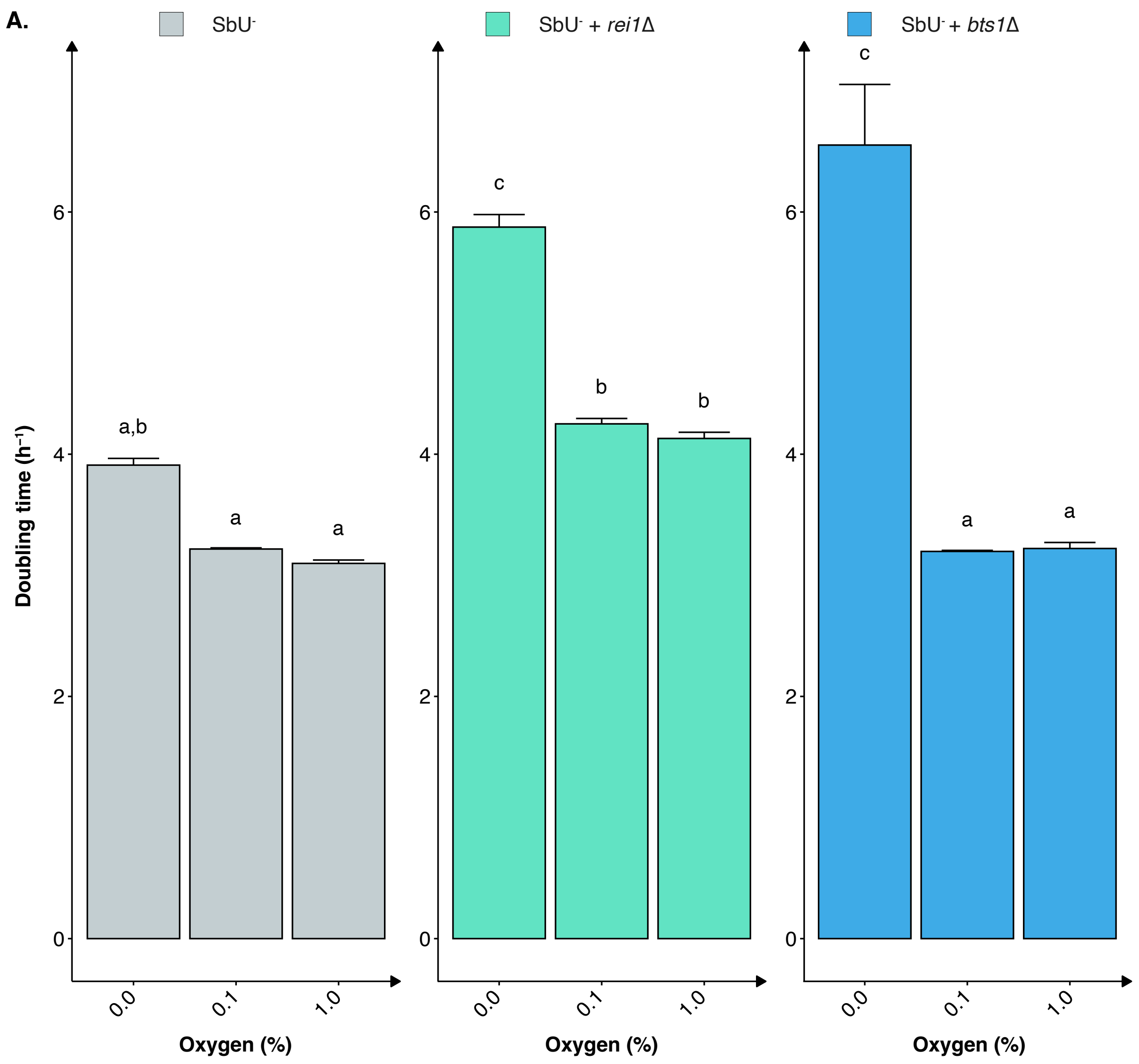

Supplementary Figure S5. Growth characterisation of the cold-sensitive strains at different oxygen concentration. (A) Bar plot of the mean doubling time (h^{-1}) under anaerobic (0 %) and microaerobic (0.1 % and 1 %) at pH 6. Data presented as mean + SEM (n = 3). Two-way ANOVA, Tukey post hoc test. The different letters (a, b, and c) above the bars indicate statistically different groups (significance level at $p < 0.05$)

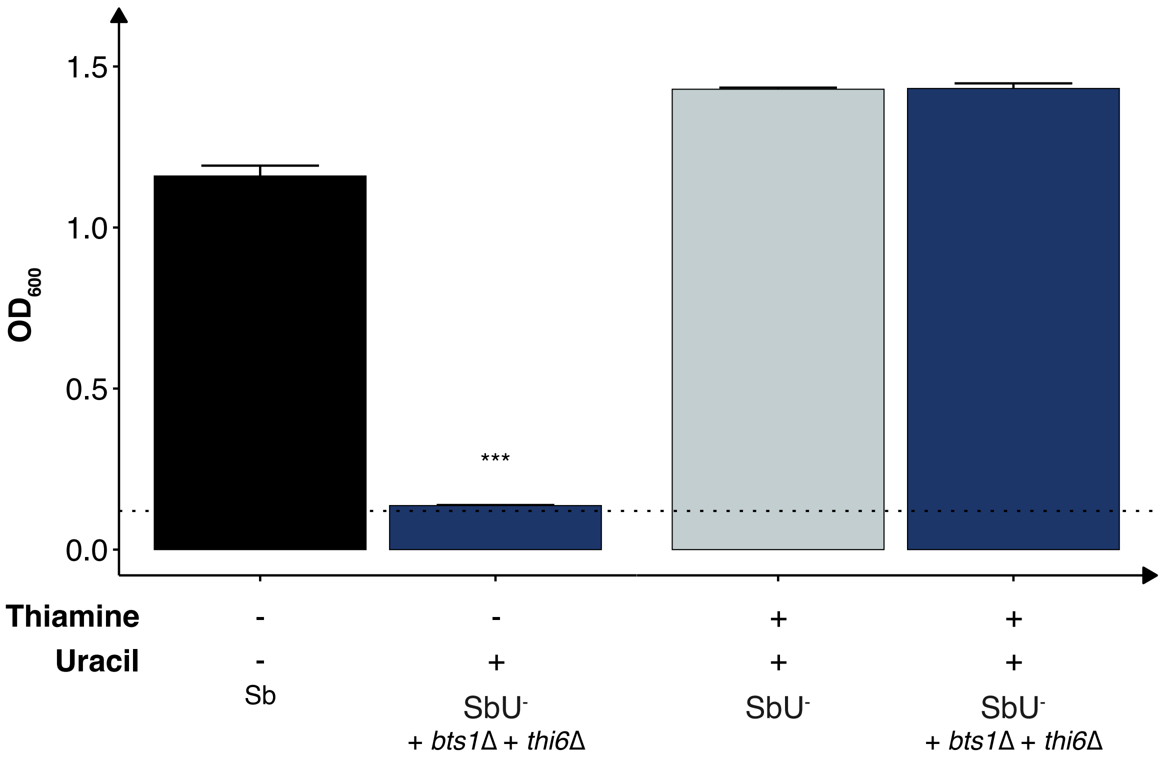

Supplementary Figure S6. Characterisation of the combined biocontainment strain. Bar plot of the mean OD_{600} after 48 hours with (+) and without (-) thiamine supplemented at 37°C. Data presented as mean + SEM (n = 3). * $p < 0.05$, ** $p < 0.01$ and *** $p < 0.001$. One-way ANOVA, Dunnett's post hoc test with SbU^- as reference.

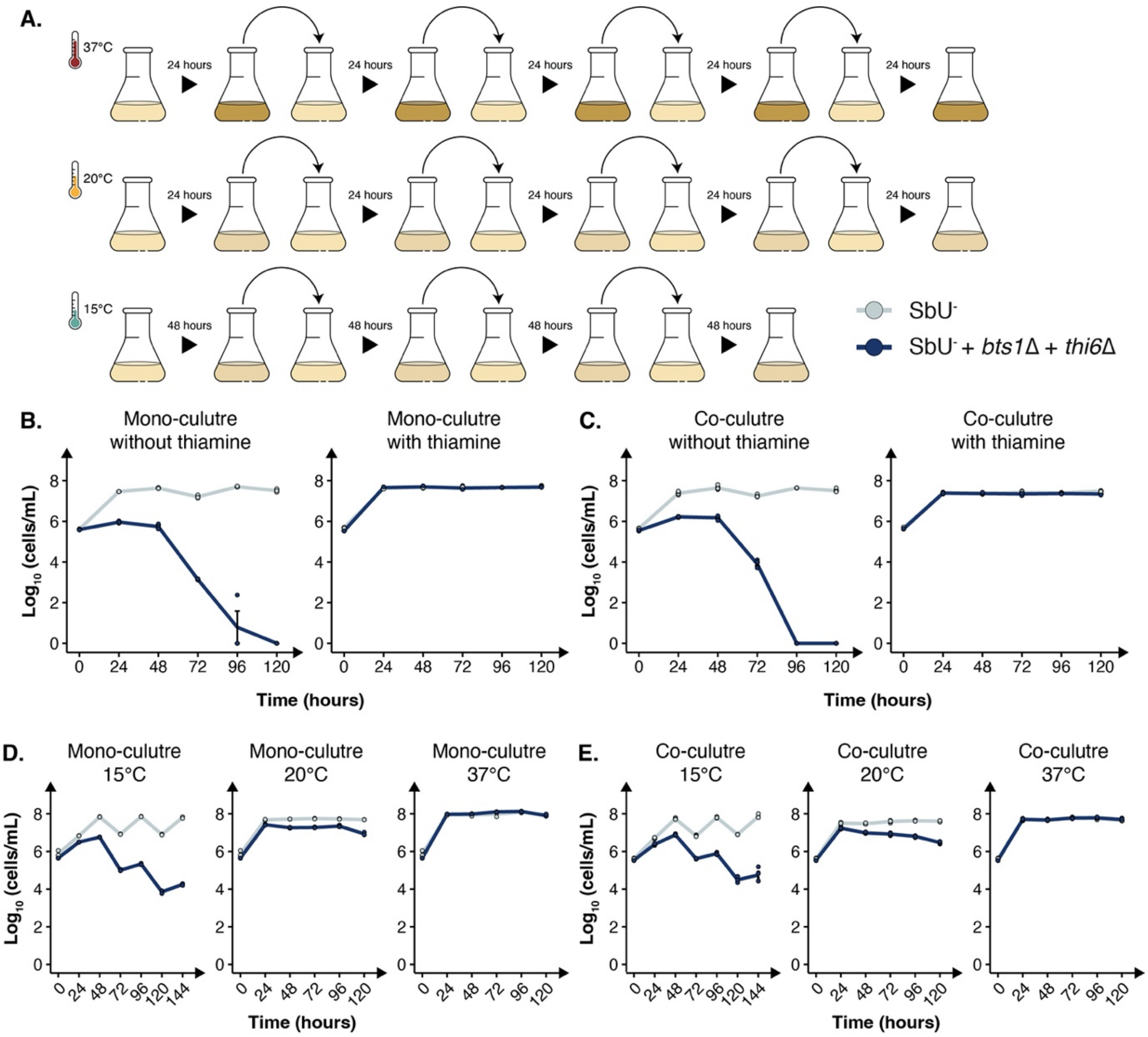

Supplementary Figure S7. Growth characterisation of the combined biocontainment strain in mono-culture and co-culture at different condition. (A) Graphical illustration of the experimental design. SbU^- and SbU^- + *bts1*Δ + *thi6*Δ were inoculated 1:1 in a culture. **(B)** Log_{10} cells/mL of SbU^- and SbU^- + *bts1*Δ + *thi6*Δ in a mono-culture and co-culture with each other in media with and without thiamine. The culture was diluted 1:100 every 48th hour for a total period of 96 hours (n = 3). **(C)** Log_{10} cells/mL of SbU^- and SbU^- + *bts1*Δ + *thi6*Δ in a mono-culture and co-culture with each other at 15°C, 20°C, and 37°C. The culture was diluted 1:100 every 24th hour for a total period of 120 hours for cultures at 20°C and 37°C, and 48th hour for a total period of 144 hours for cultures at 15°C (n = 5). Data presented as mean ± SEM.

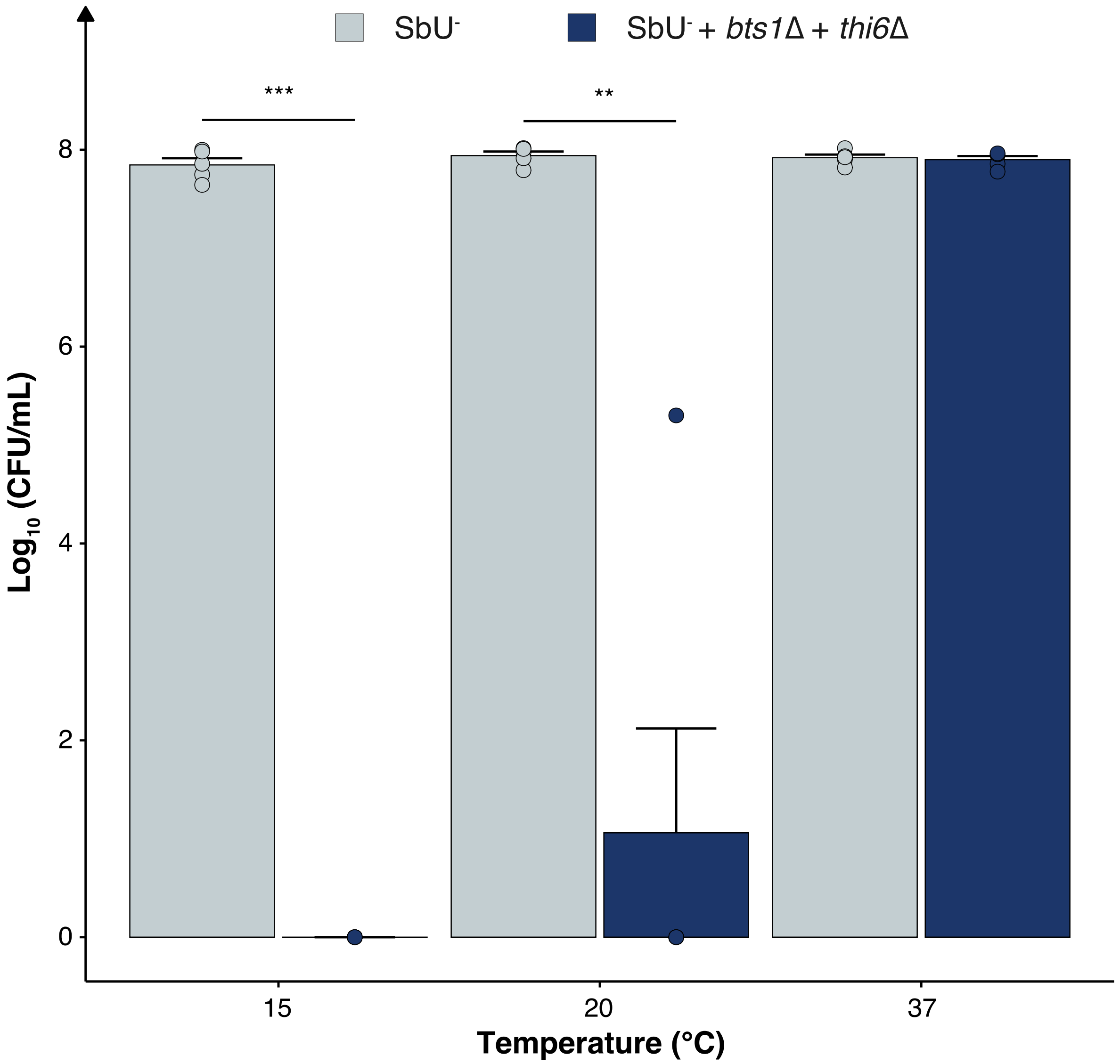

Supplementary Figure S8. Survival assay of the combined biocontainment strain. Log_{10} CFU/mL of SbU^- and SbU^- + *bts1*Δ + *thi6*Δ from a 120 hour co-culture at 37°C that was plated and incubated at 15°C, 20°C, and 37°C for 144, 96 and 48 hours, respectively. Data presented as mean + SEM (n = 5). * $p < 0.05$, ** $p < 0.01$ *** $p < 0.001$. Data was analysed with dependent sample t-test with Bonferroni adjustment for multiple comparisons.

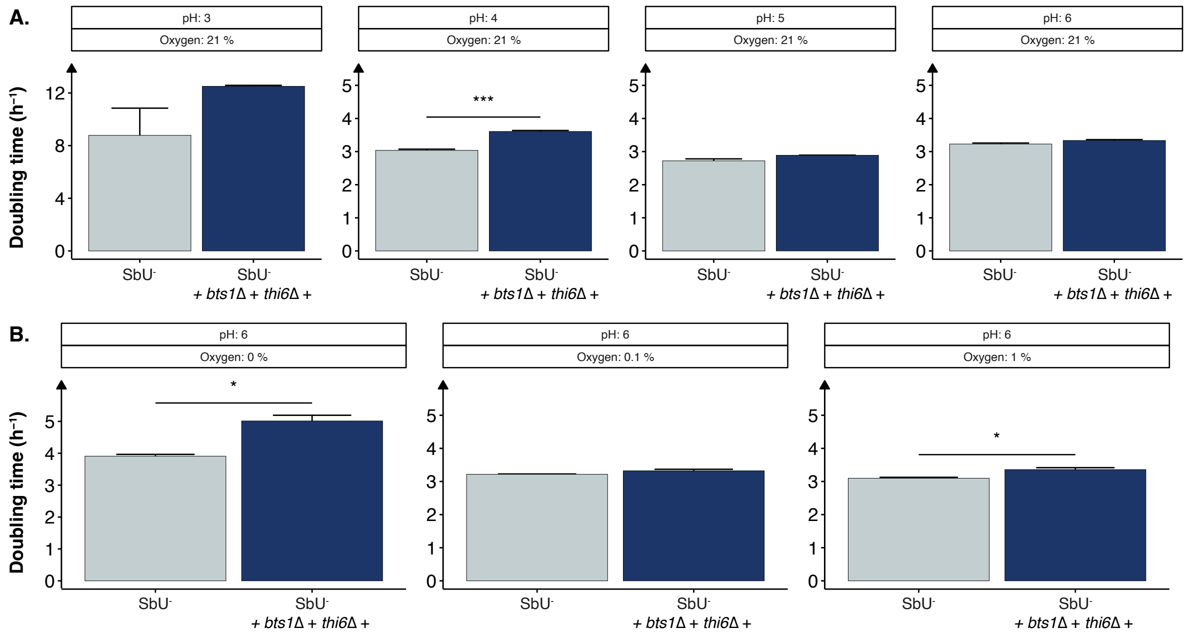

Supplementary Figure S9. Growth characterisation of the multi-layered biocontainment strain. (A) Bar plot of the mean doubling time (h^{-1}) in pH 3, 4, 5 and 6 at aerobic cultivation. **(B)** Bar plot of the mean doubling time (h^{-1}) under anaerobic (0 %) and microaerobic (0.1 % and 1 %) at pH 6. Data presented as mean + SEM (n = 3). * $p < 0.05$, ** $p < 0.01$ *** $p < 0.001$. All samples analysed with dependent sample t-test.

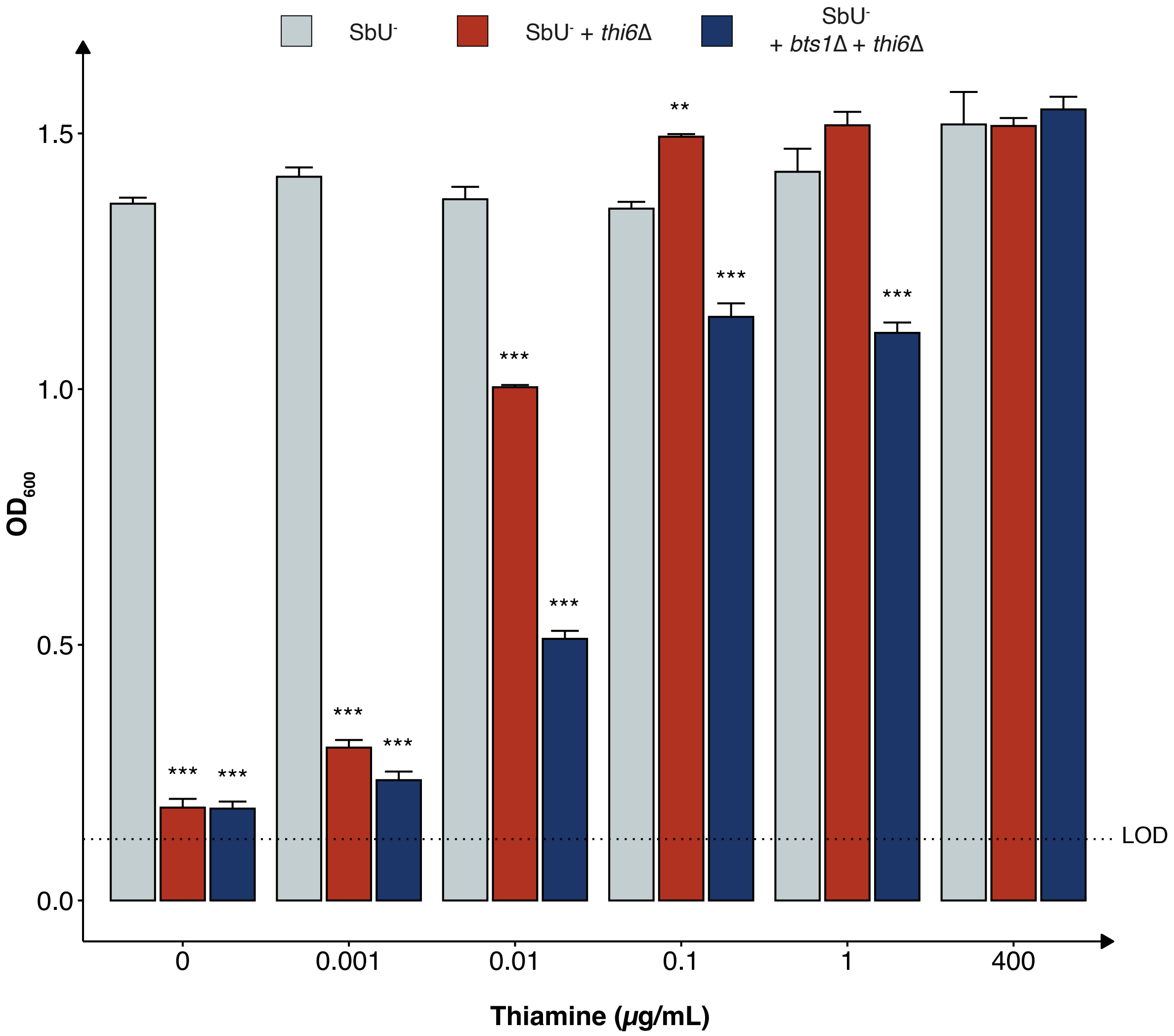

Supplementary Figure S10. Growth characterisation of the multi-layered biocontainment strain at different thiamine concentration. (A) Bar plot of mean OD_{600} after 48 hours under different concentrations of thiamine. Limit of detection (LOD). Data presented as mean + SEM (n = 3). * $p < 0.05$, ** $p < 0.01$ *** $p < 0.001$. All samples are analysed by One-way ANOVA, Dunnett's post hoc test with SbU^- as reference.

Supplementary Tables

Supplementary Table S1. Primers used in this study

Primer	Sequence	Reference
pCfB2312-gRNA_fw	atacgaagttatattaagggttg	This study
pCfB2312-gRNA_rv	taggcgtatcacgagat	This study
pCfB2312_fw	aagcttcagctgacgcgat	This study
pCfB2312-rv	tgcaggtcgacaacccttaa	This study
URA3repair_fw	tccatggagggcacagttaagccgctaaaggcattataagccaagt acaattttttactc	(Zhang et al., 2014)
URA3repair_rv	accaatgtcagcaaattttctgtcttcgaagagtaaaaaattgtacttg gcttataatgc	(Zhang et al., 2014)
HIS3repair_fw	gtaaagcgtattacaaatgaaaccaagattcagattgcgatctcttta aagggttaaccc	(Zhang et al., 2014)
HIS3repair_rv	ttctgggaagatcgagtgctctatcgctaggggttaaccctttaaaga gatcgcaatctg	(Zhang et al., 2014)
TRP1repair_fw	tccgatgctgacttgctgggtattatatgtgtgtaaaatagaaagaga acaattgacccg	(Zhang et al., 2014)
TRP1repair_rv	tacaagacttgaaattttccttgcaataaccgggtcaattgttctctttct attttacac	(Zhang et al., 2014)
URA3dg-fw	cttgcatgacaattctgcta	This study
URA3dg-rv	ttcttaacccaactgcacag	This study
HIS3dg-fw	aaagatctaccaccgctctg	This study
HIS3dg-rv	gcgattggcattatcacata	This study
TRP1dg-fw	gccgattaagaattcggtcg	This study
TRP1dg-rv	gcactgagtagtatgttgcag	This study
BTS1dg_fw	aaatcgcgaatttaccggcg	This study
BTS1dg_rv	gccgccatctctactcactc	This study
BTS1repair_fw	tcattttcaaagaagctactaatagaaagagaacaaagcgtttacga gtctggaaaatcaaataaattgatcaatcaaattagtggaggaagat agtcagaaataaagccttctctcctc	This study
BTS1repair_rv	gaggagagaaggctttatttctgactatcttcctccactaatttgattga tcaatttatttgattttccagactcgtaaacgctttgttctctttctattagt agcttctttgaaaatga	This study
REI1dg_fw	ttgattcgctgcctgttcg	This study
REI1dg_rv	cccgatattccccgtgactc	This study
REI1repair_fw	tacaggttatgagatgcttctcattagaagtcaagaagagagcatatc agtaacaatacgttctttttgcactactttttttagtattttgtcgcatatat actgcttcaccatttgtac	This study

REI1repair_rv	gtacaaatggtgaagcagtatatatgcgacaaaatactaaaaaaagtagtgcaaaaagaacgtattgttactgatatgctctcttcttgacttctaatgagaagcatctcataacctgta	This study
SNZ1dg_fw	agctttaccctggaagcacc	This study
SNZ1dg_rv	ggacgctgagagctatggac	This study
SNZ1repair_fw	agaaacctttaggaacgactagcaaatatacacagtactaatattcagttaattatcacgtttctttgaacaggtattttgagcattataacacttttcccccctcaactttgtattac	This study
SNZ1repair_rv	gtaatacaaagttgaggggggaaaaagtgttataatgctcaaaatacctgttcaaagaaacgtgataattaactgaatattagtactgtgtatatttgctagtcgttcctaaaggtttct	This study
SNO1dg_fw	ccttacccttgtcggctgag	This study
SNO1dg_rv	aagggcctgcggaagatcac	This study
SNO1repair_fw	aggttttttttcttattatttcatttcgttaaatagaaagaaaaaccatatcttaaagtaataccgccggttttccacattttatattaccaaaacctgagagatttttcacatcga	This study
SNO1repair_rv	tcgatgtgaaaaaatctctcaggttttggtaatataaaatgtggaaaaccggcggtattactttaagatatggtttttctttctatttaacgaaatgaaataataagaaaaaaaaacct	This study
THI2repair_fw	ttttcatttcatttcaccacgtatatatatagcctatatatatatccgcactagaaccaagcttattgagccttcccttcactcatttcaagaaaaaaaaagccaaaagctttgcttgga	This study
THI2repair_ rv	tccaagcaaagcttttggcttttttttcttgaaatgagtgaagggaaggctcaataagcttggttctagtgcggatatatatataggctatatatatacgtggtgaaatgaaatgaaaa	This study
THI2_dg fw	tacaatggcagccctcttgg	This study
THI2_dg rv	ccctggcagataggaaaccc	This study
THI6repair_fw	aaccatggttctcaagaccaaatcactctgaagctaaattatttaaatcaaacagcggaaactatttacaacgtaatgattttataaactgattaaataaggaagaaagcccaaaaaatt	This study
THI6repair_rv	aattttttgggctttcttccttatttaatcagtttataaaatcattacgttgtaaatagtttccgctgtttgatttaaataatttagcttcagagtgatttggtcttgagaaccatggtt	This study
THI6_dg fw	tccgtttctagctgcaggtc	This study
THI6_dg rv	agtttgttcctcctggggtg	This study
pCfB3050_fw	cttcgctattacgccagctg	This study
pCfB3050_rv	ccgattcattaatgcagctg	This study

Supplementary Table S2. Plasmids used in this study

**Plasmid name**	**Genotype**	**Marker (*E. coli / S. boulardii*)**	**Reference**
pCfB2312	CEN6_ARS4 Amp P_{TEF1}-Cas9-t_{CYC1} kanMX	Amp / KanMX	(Jessop-Fabre et al., 2016)
pCfB2312-URA3	pCfB2312; P_{SNR52}-gRNA(*URA3*)-t_{SUP4}	Amp / KanMX	This study
pCfB2312-HIS3	pCfB2312; P_{SNR52}-gRNA(*HIS3*)-t_{SUP4}	Amp / KanMX	This study
pCfB2312-TRP1	pCfB2312; P_{SNR52}-gRNA(*TRP1*)-t_{SUP4}	Amp / KanMX	This study
pCfB2909-Exe4	pCfB2909: P_{TDH3}-Exe4-t_{DIT1}**	Amp	(Hedin et al., 2022)
pCfB2909-GFP	pCfB2909: P_{TDH3}-GFP- t_{ADH1}	Amp	This study
pCfB2909-mKate	pCfB2909: P_{TDH3}-mKate-t_{ADH1}	Amp	This study
pCfB3050 (URA3)	pESC; P_{SNR52}-gRNA(XII-5)-t_{SUP4}	Amp / *URA3*	This study
pCfB3050-THI2	pESC; P_{SNR52}-gRNA(*THI2*)-t_{SUP4}	Amp / *URA3*	This study
pCfB3050-THI6	pESC; P_{SNR52}-gRNA(*THI6*)-t_{SUP4}	Amp / *URA3*	This study
pCfB3050-SNO1	pESC; P_{SNR52}-gRNA(*SNO1*)-t_{SUP4}	Amp / *URA3*	This study
pCfB3050-SNZ1	pESC; P_{SNR52}-gRNA(*SNZ1*)-t_{SUP4}	Amp / *URA3*	This study
pCfB3050-BTS1	pESC; P_{SNR52}-gRNA(*BTS1*)-t_{SUP4}	Amp / *URA3*	This study
pCfB3050-REI1	pESC; P_{SNR52}-gRNA(*REI1*)-t_{SUP4}	Amp / *URA3*	This study

Supplementary Table S3. Construct sequences used in this study

Name	Sequence	Reference(s)
P_{TDH3}	cagttcgagtttatcattatcaatactgccatttcaaagaatacgtaaataattaa tagtagtgattttcctaactttatttagtcaaaaaattggccttttaattctgctgta acccgtacatgcccaaaatagggggcgggttacacagaatatataacatcat aggtgtctgggtgaacagtttattcctggcatccactaaatataatggagccc gctttttaagctggcatccagaaaaaaaaagaatcccagcaccaaaatattgt tttcttcaccaaccatcagttcataggtccattctcttagcgcaactacacagaa caggggcacaaacaggcaaaaaacgggcacaacctcaatggagtgatgc aacctgcttggagtaaatgatgacacaaggcaattgacctacgcatgtatcta tctcattttcttacaccttctattaccttctgctctctctgatttggaaaaagctgaa aaaaaaggttgaaaccagttccctgaaattattcccctatttgactaataagtat ataaagacggtaggtattgattgtaattctgtaaatctatttcttaaacttcttaaa ttctacttttatagttagtcttttttttagttttaaaacactaagaacttagtttcgaat aaacacacataaacaaacaaa	This study
t_{ADH1}	gtagatacgttgttgacacttctaaataagcgaatttcttatgatttatgatttttat tattaaataagttataaaaaaaataagtgtatacaaattttaaagtgactcttagg ttttaaaacgaaaattcttattcttgagtaactctttcctgtaggtcaggttgctttc tcaggtatagcatgaggtcgctc	This study
mKate	atggtttctgaactcatcaaggaaaacatgcacatgaaactttacatggaagg tactgtgaacaatcatcattttaagtgtacatccgagggtgaaggcaaacctta cgaaggaactcaaactatgagaattaaagctgtagaaggtggaccattacct tttgcatttgatatcttggcaacatcattcatgtatgggagcaagacattcataa accatactcaaggtataccagactttttcaaacagagttttccagagggttttac atgggaaagagtaacaacgtacgaggatggaggtgtattgacagccactca agacacatcacttcaagatgggtgtttaatctacaatgtcaagattagaggcg tcaatttcccttctaatggtccagttatgcagaaaaagacattaggctgggaa gcgtcaaccgaaaccctgtaccctgctgatggtggcctagaaggcagagct gacatggcccttaaactggttggtggagggcatctaatctgcaatttgaaaac cacttatcgttctaaaaagccagccaaaaacctaaagatgccaggtgtttact acgtcgaccgaagattagaaaggattaaagaggctgataaagagacttatgt tgaacaacacgaagtggcagtggctagatactgtgatttgccatctaagttgg gacacagataa	This study
yEGFP	atgtctaaaggtgaagaattattcactggtgttgtcccaattttggttgaattaga tggtgatgttaatggtcacaaattttctgtctccggtgaaggtgaaggtgatgc tacttacggtaaattgaccttaaaatttatttgtactactggtaaattgccagttcc atggccaaccttagtcactactttcggttatggtgttcaatgttttgcgagatac ccagatcatatgaaacaacatgactttttcaagtctgccatgccagaaggttat gttcaagaaagaactatttttttcaaagatgacggtaactacaagaccagagc tgaagtcaagtttgaaggtgataccttagttaatagaatcgaattaaaaggtatt gattttaaagaagatggtaacattttaggtcacaaattggaatacaactataact ctcacaatgtttacatcatggctgacaaacaaaagaatggtatcaaagttaact tcaaaattagacacaacattgaagatggttctgttcaattagctgaccattatca	This study

	acaaaatactccaattggtgatggtccagtcttgttaccagacaaccattactt atccactcaatctgccttatccaaagatccaaacgaaaagagagaccacatg gtcttgttagaatttgttactgctgctggtattacccatggtatggatgaattgta caaatga	
Exendin-4	tctaccaacggaatgcgtgcgatcgcgtgcattccgagtttatcattatcaata ctgccatttcaaagaatacgtaaataattaatagtagtgattttcctaactttattt agtcaaaaaattagccttttaattctgctgtaacccgtacatgcccaaaatagg gggcgggttacacagaatatataacatcgtaggtgtctgggtgaacagtttat tcctggcatccactaaatataatggagcccgctttttaagctggcatccagaa aaaaaaagaatcccagcaccaaaatattgttttcttcaccaaccatcagttcat aggtccattctcttagcgcaactacagagaacaggggcacaaacaggcaa aaaacgggcacaacctcaatggagtgatgcaacctgcctggagtaaatgat gacacaaggcaattgacccacgcatgtatctatctcattttcttacaccttctatt accttctgctctctctgatttggaaaaagctgaaaaaaaaggttgaaaccagtt ccctgaaattattcccctacttgactaataagtatataaagacggtaggtattga ttgtaattctgtaaatctatttcttaaacttcttaaattctacttttatagttagtcttttt tttagttttaaaacaccaagaacttagtttcgaataaacacacataaacaaaca aaaacaaaatgagatttccatctatttttactgctgttttgtttgctgcttcttctgc tttggctgctccagttaatactactactgaagatgaaactgctcaaattccagct gaagctgttattggttattctgatttggagggtgactttgatgttgctgttttgcca ttttctaactctactaacaacggtttgctattcatcaacactactatcgcttctatc gctgctaaagaagaaggtgtttctttggataaaagagaagaaggtgaaccaa aacatggtgaaggcacattcacatctgatctgtccaaacaaatggaggagg aagcggtacgtttatttattgaatggttaaaaaacgggggacctagctccggc gcgccccccccgagctaataaagtaagagcgctacattggtctaccttttttctt ttacttaaacattagttagttcgttttctttttctttttttatgtttccccccccaaagttc tgattttataatattttatttcacacaattccatttaacagaggggggaatagattct ttagcttagaaaattagtgatcaatatatatttgcctttcttttcatcttttcagtgat attaatggtttcgagacactgcaatggccctactagtgctgaggcattaat	(Hedin et al., 2022)

Supplementary Table S4. gRNA used in this study

gRNA	Sequence	Reference
URA3	gagtaaaaaattgtacttgg	(Zhang et al., 2014)
HIS3	ccctttaaagagatcgcaat	(Zhang et al., 2014)
TRP1	gtcaattgttctctttctat	(Zhang et al., 2014)
THI2	actacaattatctccatgtt	This study
THI6	ttaaataaccataaaatgaa	This study
SNO1	gacgccttaattattcccgg	This study
SNZ1	acccaactgcattaacaatg	This study

BTS1	ttgctgaggacattacagag	This study
REI1	gaagatgactgggaagacgt	This study

Table S5. Strains used in this study

Strain	Genotype	Marker	Parental strain	Reference
Sb		N/A	SB-ATCC-796	This study
SbU$^-$	Sb *URA3*^{S81X}	N/A	Sb	This study
SbH$^-$	Sb *HIS3*^{G26X}	N/A	Sb	This study
SbT$^-$	Sb *TRP1*^{P12X}	N/A	Sb	This study
SbU$^-$ + *HIS3*^{G26X}	Sb *URA3*^{S81X} + *HIS3*^{G26X}	N/A	SbU$^-$	This study
SbH$^-$ + *TRP1*^{P12X}	Sb *HIS3*^{G26X} + *TRP1*^{P12X}	N/A	SbH$^-$	This study
SbU$^-$ + *HIS3*^{G26X} + *TRP1*^{P12X}	Sb *URA3*^{S81X} + *HIS3*^{G26X}	N/A	SbU$^-$ + *HIS3*^{G26X}	This study
SbU$^-$ + *thi6*Δ	Sb *URA3*^{S81X} + *thi6*Δ	N/A	SbU$^-$	This study
SbU$^-$ + *thi2*Δ	Sb *URA3*^{S81X} + *thi2*Δ	N/A	SbU$^-$	This study
SbU$^-$ + *sno1*Δ	Sb *URA3*^{S81X} + *sno1*Δ	N/A	SbU$^-$	This study
SbU$^-$ + *snz1*Δ	Sb *URA3*^{S81X} + *snz1*Δ	N/A	SbU$^-$	This study
SbU$^-$ + *sno1*Δ + *snz1*Δ	Sb *URA3*^{S81X} + *sno1*Δ + *snz1*Δ	N/A	SbU$^-$+ *sno1*Δ	This study
SbU$^-$ + *rei1*Δ	Sb *URA3*^{S81X} + *rei1*Δ	N/A	SbU$^-$	This study
SbU$^-$ + *bts1*Δ	Sb *URA3*^{S81X} + *bts1*Δ	N/A	SbU$^-$	This study
SbU$^-$ + *bts1*Δ + *thi6*Δ	Sb *URA3*^{S81X} + *bts1*Δ + *thi6*Δ	N/A	SbU$^-$+ *bts1*Δ	This study
(SbU$^-$)-Exe4	SbU$^-$ + XII-5 P$_{TDH3}$-Exe4-t$_{DIT1}$**	N/A	SbU$^-$	(Hedin et al., 2022)
(SbU$^-$ + *thi6*Δ)-Exe4	SbU$^-$ + *thi6*Δ + XII-5 P$_{TDH3}$-Exe4-t$_{DIT1}$**	N/A	SbU$^-$ + *thi6*Δ	This study
(SbU$^-$ + *bts1*Δ)-Exe4	SbU$^-$ + *bts1*Δ + XII-5 P$_{TDH3}$-Exe4-t$_{DIT1}$**	N/A	SbU$^-$+ *bts1*Δ	This study
(SbU$^-$ + *bts1*Δ + *thi6*Δ)-Exe4	SbU$^-$ + *bts1*Δ + *thi6*Δ + XII-5 P$_{TDH3}$-Exe4-t$_{DIT1}$**	N/A	SbU$^-$ + *bts1*Δ + *thi6*Δ	This study
(SbU$^-$)-GFP	SbU$^-$ + XII-5 P$_{TDH3}$-yEGFP-t$_{ADH1}$	N/A	SbU$^-$	This study
(SbU$^-$ + *bts1*Δ + *thi6*Δ)-mKate	SbU$^-$ + *bts1*Δ + *thi6*Δ + XII-5 P$_{TDH3}$-mKate-t$_{ADH1}$	N/A	SbU$^-$ + *bts1*Δ + *thi6*Δ	This study

Reference

Hedin, K. A., Zhang, H., Kruse, V., Rees, V. E., Bäckhed, F., Greiner, T. U., Vazquez-Uribe, R., & Sommer, M. O. A. (2022). Oral delivery of GLP-1R agonist by an engineered probiotic yeast strain has anti-obesity effects in mice. *BioRxiv*, [Preprint]. https://doi.org/10.1101/2022.12.21.521368

Jessop-Fabre, M. M., Jakočiūnas, T., Stovicek, V., Dai, Z., Jensen, M. K., Keasling, J. D., & Borodina, I. (2016). EasyClone-MarkerFree: A vector toolkit for marker-less integration of genes into Saccharomyces cerevisiae via CRISPR-Cas9. *Biotechnology Journal*, *11*(8), 1110–1117. https://doi.org/10.1002/BIOT.201600147

Zhang, G. C., Kong, I. I., Kim, H., Liu, J. J., Cate, J. H. D., & Jin, Y. S. (2014). Construction of a quadruple auxotrophic mutant of an industrial polyploid Saccharomyces cerevisiae strain by using RNA-guided Cas9 nuclease. *Applied and Environmental Microbiology*, *80*(24), 7694–7701. https://doi.org/10.1128/AEM.02310-14/SUPPL_FILE/ZAM999105872SO1.PDF
